# Transduction of Pig Small Airway Epithelial Cells and Distal Lung Progenitor Cells by AAV4

**DOI:** 10.3390/cells10051014

**Published:** 2021-04-25

**Authors:** Oliver G. Chen, Steven E. Mather, Christian M. Brommel, Bradley A. Hamilton, Annie Ehler, Raul Villacreses, Reda E. Girgis, Mahmoud Abou Alaiwa, David A. Stoltz, Joseph Zabner, Xiaopeng Li

**Affiliations:** 1 Department of Pediatrics & Human Development, Michigan State University, Grand Rapids, MI 49503, USA; chengua5@msu.edu; 2 Department of Internal Medicine, University of Iowa, Iowa City, IA 52242, USA; steven-mather@uiowa.edu (S.E.M.); christian-brommel@uiowa.edu (C.M.B.); bradley5@stanford.edu (B.A.H.); annie.ehler.4@gmail.com (A.E.); raul-villacresesrada@uiowa.edu (R.V.); mahmoud-aboualaiwa@uiowa.edu (M.A.A.); david-stoltz@uiowa.edu (D.A.S.); 3 Pulmonary Medicine, Spectrum Health, Grand Rapids, MI 49503, USA; reda.girgis@spectrumhealth.org; 4 Department of Biomedical Engineering, University of Iowa, Iowa City, IA 52242, USA; 5 Department of Molecular Physiology and Biophysics, Carver College of Medicine, University of Iowa, Iowa City, IA 52242, USA

**Keywords:** cystic fibrosis 1, CFTR, small airway epithelia, progenitor cells, AAV4

## Abstract

Cystic fibrosis (CF) is caused by genetic mutations of the CF transmembrane conductance regulator (CFTR), leading to disrupted transport of Cl^−^ and bicarbonate and CF lung disease featuring bacterial colonization and chronic infection in conducting airways. CF pigs engineered by mutating CFTR develop lung disease that mimics human CF, and are well-suited for investigating CF lung disease therapeutics. Clinical data suggest small airways play a key role in the early pathogenesis of CF lung disease, but few preclinical studies have focused on small airways. Efficient targeted delivery of CFTR cDNA to small airway epithelium may correct the CFTR defect and prevent lung infections. Adeno-associated virus 4 (AAV4) is a natural AAV serotype and a safe vector with lower immunogenicity than other gene therapy vectors such as adenovirus. Our analysis of AAV natural serotypes using cultured primary pig airway epithelia showed that AAV4 has high tropism for airway epithelia and higher transduction efficiency for small airways compared with large airways. AAV4 mediated the delivery of CFTR, and corrected Cl^−^ transport in cultured primary small airway epithelia from CF pigs. Moreover, AAV4 was superior to all other natural AAV serotypes in transducing ITGα6β4^+^ pig distal lung progenitor cells. In addition, AAV4 encoding eGFP can infect pig distal lung epithelia in vivo. This study demonstrates AAV4 tropism in small airway progenitor cells, which it efficiently transduces. AAV4 offers a novel tool for mechanistical study of the role of small airway in CF lung pathogenesis in a preclinical large animal model.

## 1. Introduction

Cystic fibrosis (CF) is caused by genetic mutations of the CF transmembrane conductance regulator (CFTR), leading to disrupted transport of Cl^−^ and bicarbonate [1,2]. CF lung disease manifests as chronic airway infection and inflammation, the leading causes of CF morbidity and mortality. Though CFTR potentiators and correctors have been approved to treat CF patients with certain CFTR defects [3], new therapeutic strategies are still urgently needed for CF patients not responsive to current therapy. The CF pig model, engineered with deletion or mutation of CFTR, develops pathologies that mimic human CF [4], including spontaneous airway infections [4]. We have utilized the CF pig model for proof-of-concept gene therapy studies and for understanding the regional differences in airway epithelium [5,6].

Clinical data suggest small airways are of major importance in early CF pathogenesis. For example, histopathological samples from CF infants show bronchiolar dilation, thickening of the wall of small airways, and air trapping in the alveolar space [7,8,9,10,11]. Pulmonary function tests of young people with CF (6–16 years old) show progressive decline in small airway function as measured by Forced Expiratory Flow (FEF_25–75%_), whereas large airway function measured by Forced Expiratory Volume in 1 s (FEV1) remains stable [12]. In older people with CF, compared to older people with Chronic Obstructive Pulmonary Disease (COPD), small airways are nearly 300% thicker, whereas large airways have only 10% greater thickness [13]. These data suggest small airways play a key role in the early pathogenesis of CF lung diseases [7,8,9,10,11]. Most laboratory studies of CF lung disease, however, have focused on the larger central airways, rather than the small airways where the disease may initiate. Therefore, potentially druggable targets to prevent CF progression have been overlooked.

Efficient gene therapy can conceptually correct the CFTR defect and prevent lung infections. Several types of viral vectors have been used to transfer functional CFTR genes into the lungs of CF ferrets and CF pigs [14,15]. Recombinant adeno-associated virus (AAV) has emerged as an attractive delivery tool for gene transfer approaches due to its non-pathogenic and less immunogenic safety profile, ability to transduce dividing and non-dividing cells, and tissue and species specificity [16,17,18]. The efficiency of AAV targeting airway epithelia has significantly improved [19,20,21,22,23,24] via mutagenesis of AAV capsids [25] and targeted evolution selection of efficient serotypes [17,26]. Our group previously reported a targeted evolution selection process in primary cultures of human airway epithelia to yield a novel AAV2.5T. Unfortunately, AAV2.5T does not transduce pig airway epithelia [27]. We applied a novel in vivo evolution strategy to select an AAV capsid (AAV2H22) that efficiently infects pig airway epithelia but does not transduce pig alveolar epithelial cells in vitro [5]. AAV4 is a natural AAV serotype rarely tested for CF gene therapy, as one previous study showed that mucins inhibit AAV4 transduction of cultured human large airway epithelial cells [28]. This is presumably because mucins glycosylated with α2-3 O-linked sialic acids bind to AAV4 and prohibit AAV4 from transfecting epithelial cells [28]. However, after intravenous administration, AAV4 targets the lungs more than other organs in mice [29,30], although the cell types transduced by AAV4 in the lungs are not clear.

For gene therapy to be a viable therapeutic strategy for CF, it is necessary to target the appropriate cell type. Targeting progenitor cells rather than terminally differentiated cells is a strategy to achieve durable transgene expression in the lung. However, discrete populations of epithelial progenitor cells, with distinct differentiation potentials, vary along the proximal to distal axis of the airways [31,32]. We have focused on identifying progenitor cells residing in the distal small airways, where clinical and autopsy observations suggest CF originates. In previous studies, we identified integrin (ITG) α6β4^+^ cells with multipotent stem/progenitor potential for both small airway and alveolar epithelia in the human and mouse lung [33,34]. We term these cells distal lung epithelial progenitors (DLEP). DLEPs have the potential to differentiate into multiple lineages, including K5^+^ basal cells, CC10^+^ club cells and Muc5AC^+^ goblet cells in humans and CC10^+^ club, SPC^+^ type II alveolar, and T1α^+^ type I alveolar cells in mice [33,34]. Moreover, DLEPs can regenerate the lung in a mouse model of injury [33,34].

Our objective, herein, was to identify AAV vectors that target DLEPs in the pig lung. We selected DLEPs as our initial focus because of our interest in the role of the small airway in the pathogenesis of CF and the cuboidal epithelia of small airway allowing vector access from the apical side, as well as the ability of these cells to proliferate and differentiate into the various lineages of airway and alveolar epithelial cells. Our screen of natural and evolved AAV serotypes revealed extremely high tropism of AAV4 for pig DLEPs.

## 2. Materials and Methods

### 2.1. Animals

All animal studies were reviewed and approved by the Michigan State University and University of Iowa Animal Care and Use Committee. Non-CF pigs and CF pigs within one week old were obtained from Exemplar Genetics (Exemplar Genetics, Sioux Center, IA, USA) and Michael Fanning Farms (Howe, IN, USA).

### 2.2. Isolation of DLEPs

A schematic workflow for isolation of large, small airway epithelial cells and DLEPs was listed in supplementary Figure S1. First, airways were detached from lung parenchyma by blunt dissection. Then small airways were further separated from large airways by microdissection. Large, small airways cells and distal lung progenitor cells were isolated from different regions of the pig lungs. DLEPs were isolated from the pig distal lung parenchymal tissue using a modified protocol [33]. Briefly, the pulmonary artery was perfused with PBS solution and the distal air spaces were lavaged 10 times with Ca^2+^-and Mg^2+^-free PBS solution (0.5 mM EGTA and 0.5 mM EDTA). Piglet lungs were excised and the whole airway tree was micro-dissected by carefully combing off the parenchymal tissue. A trypsin-elastase combination (0.5 mg/mL elastase in 0.5% trypsin solution) was used to enzymatically digest the parenchymal tissue at 37 °C for 60 min with shaking. Single-cell suspensions were co-stained with a rat monoclonal α6 antibody (BD Biosciences, San Jose, CA, USA), a mouse monoclonal primary antibody against EPCAM, and an antibody against CD31 followed by secondary antibodies anti-rat Alexa-Fluor 647 and anti-mouse Alexa-Fluor 488 (Invitrogen, Grand Island, NY, USA). Cells were sorted for α6^+^/EPCAM^+^/CD31^−^ expression using the FACSAria III Cell Sorter (BD Biosciences) at the University of Iowa Flow Cytometry Core Facility.

### 2.3. Isolation of Large and Small Airway Epithelia

Piglet lungs were excised and the whole airway tree was micro-dissected by carefully combing off the parenchymal tissue. Subsequently, the vascular tissue was separated from the airway tree by blunted dissection. Proximal large airways, including trachea and main stem bronchi, and distal small airways (diameter ~200 µm) were dissected out separately from the airway tree. In order to isolate enough small airway epithelia for study in vitro, the entire airway tree was micro-dissected. Next, primary porcine airway epithelia were isolated according to an adapted procedure originally developed for tracheal airway cells [35]. Primary epithelial cells were seeded onto collagen-coated, semi-permeable membranes (Corning #3470) at density of 10^6^ cells/cm^2^ and cultured at the air-liquid interface at 37 °C in a 5% CO_2_ atmosphere, as previously described [36]. For small airway epithelia, the typical yield per lung was 3–4 × 10^6^ cells. In the first week of seeding at the air-liquid interface, cells were maintained in Small Airway Growth Media (Lonza, Basel, Switzerland) supplemented with 10 ng/mL keratinocyte growth factor (KGF) for one week, after which cells were maintained in USG media. All experiments were performed ~2 weeks after seeding on matched large and small airway epithelia isolated from the same animal and cultured under identical conditions.

### 2.4. Viral Transduction of Pig Airway Epithelia

The self-complimentary AAV vectors utilized in this study expressed GFP under the control of a CMV promoter. AAV2 or AAV2H22 derivatives have been previously reported [5]. AAV production was performed as a fee for service at the University of Iowa Viral Vector Core (https://medicine.uiowa.edu/vectorcore/, accessed on 20 April 2021). CFTR transgene in AAV4 contains a deleted portion of the R domain (CFTRΔR, 708−759), a shortened CMV immediate/early (173CMVie) enhancer/promoter, and minimal poly(A) signal as described by Ostedgaard et al. [37].

For transduction of well-differentiated primary airway epithelia cultured at the air-liquid interface (ALI) in vitro, cells were pre-treated treated with 5 µM doxorubicin (Calbiochem, La Jolla, CA, USA) for 4 h, which has been shown to effectively increase viral capsid ubiquitination and transduction of recombinant AAV vectors [38]. Next, AAV vectors harboring eGFP (10^5^ vg/cell) were diluted in EMEM and added to the apical surface of pig airway epithelia. Samples were incubated overnight at 37 °C, followed by 5 µM Hoechst-33342 (#H1399, Invitrogen, Carlsbad, CA, USA) for 4 h, as previously described [39]. Then, two weeks after AAV transduction, cells were analyzed for eGFP expression.

Freshly isolated pig DLEPs (4 × 10^4^ cells/transwell) were mixed with the AAV serotypes (10^5^ vg/cell) and cultured in 100 µL Matrigel/ DMEM (1:1) per transwell. Then, two weeks later, GFP^+^ positive colonies were visualized and counted as previously described [33].

For transduction in vivo, AAV4 > eGFP (1 × 10^12^ vg/animal) was instilled into the lungs of one-week old non-CF pigs (4–5 kg). After intubating the pigs, a PE50 catheter was used to guide delivery to the left distal lung. The virus was administered in the presence of 250 μM doxorubicin in 0.5 mL solution. Then, two weeks post-infection, animals were euthanized by intracardiac injection of Euthasol and serial sections of the whole airway tree analyzed for GFP expression as described below.

### 2.5. Fluorescence Imaging and Immunofluorescence

For in vitro analysis of GFP expression, cells were fixed with 4% paraformaldehyde. For in vivo analysis of GFP expression, tissues were fixed with 4% paraformaldehyde, embedding in O.C.T. compound (Tissue Tek by Sakura Finetek, Torrance, CA, USA), cryosectioned into 7 μm sections, and permeabilized in 0.2% Triton X-100. Nuclei were counterstained with DAPI. GFP-positive cells visualized with an Olympus Fluoview FV1000 confocal microscope with a UPLSAPO ×60 oil lens.

### 2.6. Lectin Profiling

Fluorescein-conjugated lectins were purchased from Vector Laboratories (Burlingame, CA, USA). The lectins were bound to cells on ice for 15 min at the following concentrations: Concanavalin A (ConA), 1 mg/mL; WGA, 30 μg/mL, jacalin, 1 mg/mL; MMA Maakia amurensis lectin I, 100 μg/mL. Next, the cells were washed three times with ice-cold phosphate-buffered saline (PBS). The cells were then fixed with 4% paraformaldehyde in PBS at room temperature for 25 min, followed by another PBS wash. Samples were then visualized using an Olympus IX71 fluorescence microscope.

### 2.7. Ussing Studies

The short-circuit current (Isc) was measured, using a Cl^−^ gradient in modified Ussing chambers (Physiological Instruments) as previously described [6].

### 2.8. Statistics

Data are expressed as mean ±SEM. For analyses that compared large and small airways from the same animal, we used a non-parametric Wilcoxon signed-rank test. *p* values are presented in figure legends. All analyses were done using Prism software Version 8.0.

## 3. Results

### 3.1. DLEPs Can Be Isolated from Pig Distal Lungs 

**Figure 1:**
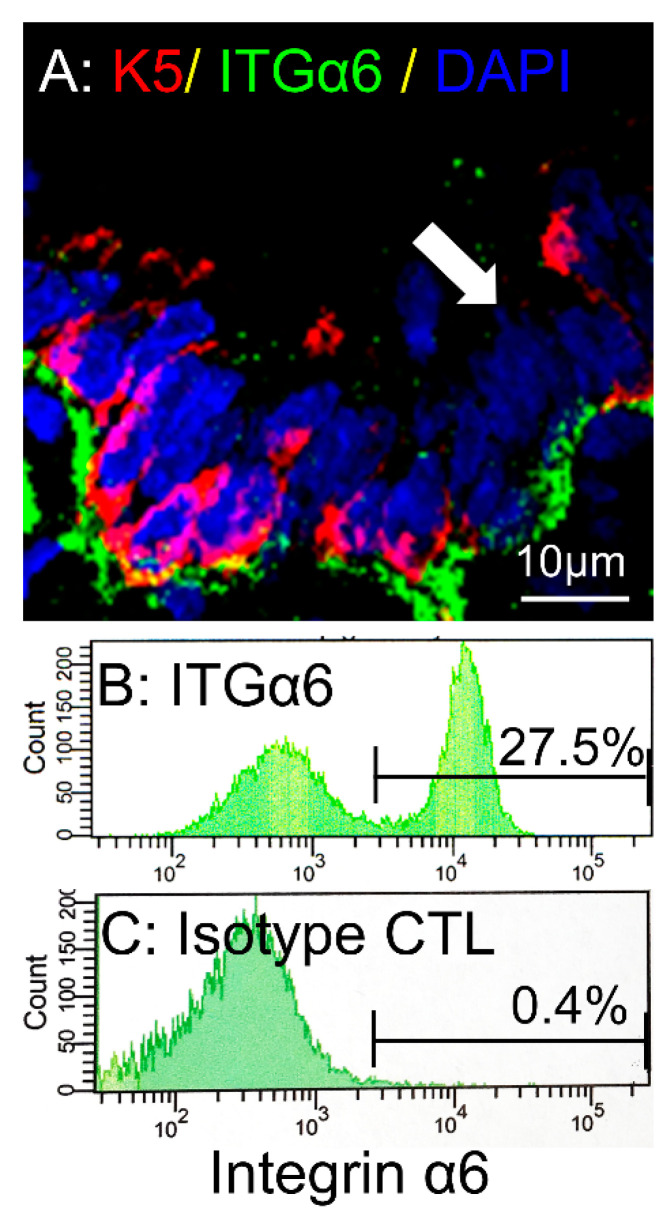
Identification of integrin (ITG) α6^+^ cells in the pig distal lung. (**A**): Pig distal lung was immunostained with Keratin 5 (K5, red), integrin (ITG) α6 (ITGa6, green), and DAPI (blue). Arrow indicates that ITG α6^+^ progenitor cells (green) are adjacent to K5^+^ (red) basal cells. (**B**): FACS assay for immunostained ITGα6^+^ cells among cultured small airway epithelial cells indicated that 27.5% of the cells are ITGα6+ basal cells. (**C**): FACS assay for isotype control antibody immunostained cells among cultured small airway epithelial cells. All the cells came from non-CF pigs.

**Figure 2:**
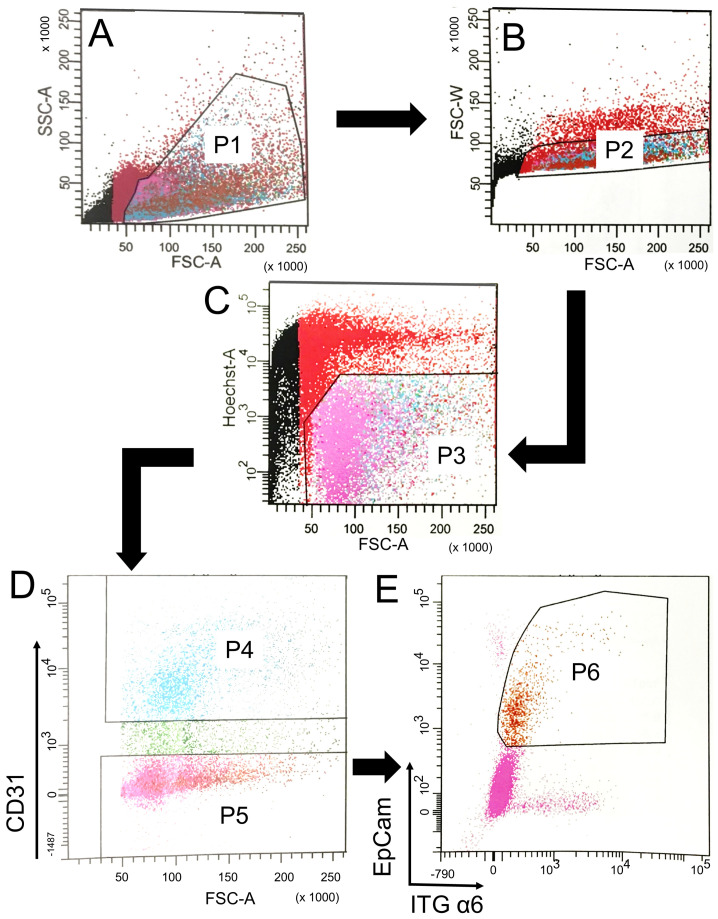
Isolation of ITG α6^+^/EPCAM^+^/CD31^−^ progenitor cells in the pig distal lung. The surface expression of ITG α6, EPCAM and CD31 of single cells preparation isolated from a wide type pig lung was analyzed by FACS. (**A**) SSC vs. FSC density plot. P1 was gated for next step analysis. (**B**) FSC-Width vs. FSC-Area density plot. P2 was gated to exclude doublets for next step analysis (**C**) Hoechst 33258 was used to stain dead cells. P3 was gated to select live single cells for next step analysis. (**D**) CD31^+^ endothelial cells were excluded by P4 gate using FASC strategy. P5 was gated for next step analysis. Cells positive for both ITG α6 and EPCAM were identified as progenitor cells using P6 gate strategy (**E**). Black arrows indicate sequential workflow. All the cells came from non-CF pigs.

We previously developed a method to selectively isolate the different regions of the pig airway: large airways, small airways, and distal parenchyma (Figure S1) [6]. Large airway epithelial cells are isolated from trachea and bronchus. Small airway epithelial cells are isolated from terminal airways with a diameter <200 μm [6]. DLEPs are isolated from the distal parenchyma using α6 ITG, a marker which was previously used for identifying DLEPs in human and mice [33,34]. We immunostained pig small airways and distal parenchyma with antibodies against α6 ITG and demonstrated that, similar to human small airway, antibodies against α6 ITG labeled both K5^+^ basal cells and the putative K5^−^α6β4^+^ DLEPs (Figure 1A). We also verified that the antibody, raised against human α6 ITG, could recognize pig α6 ITG (Figure 1B,C) for fluorescence-activated cell sorting (FACS). We next immunostained single cell preparations from enzyme digested small airways and distal lung parenchyma for α6 ITG (progenitor cell marker), EPCAM (epithelial marker) and CD31 (endothelial marker). DLEPs were isolated using FACS to sort for ITG α6^+^/EPCAM^+^ (epithelial marker)/CD31^−^ (endothelial marker) cells (Figure 2). These results demonstrated the feasibility of isolating DLEPs from pig lung.

### 3.2. Screening Reveals AAV Serotypes with High Transduction Efficiency for Pig DLEPs 

We serially screened both naturally occurring and novel AAV serotypes encoding eGFP to identify those which would best transduce pig DLEPs. We recently identified a novel AAV serotype, AAV2H22, which has high tropism for pig large and small airways, using a directed evolution technique [5,17]. AAV2H22 is identical to AAV2 except for five amino acid mutations in AAV2H22 (E67A, S207G, Q598L, I648V, and V708I) [5]. We screened AAV2H22 and five AAV2H22 derivatives, each with an individual back-mutation reverting their sequences to that of AAV2 at either A67E, G207S, L598Q, V648I, or I708V, for transduction efficiency on DLEPs. Conversely, we screened AAV2 and five AAV2 derivatives, each with one of the point-mutations (E67A, S207G, Q598L, I648V, and V708I) added to the AAV2 sequence. The DLPEs formed colonies from a single cell in the Matrigel 2 weeks after seeding. Figures S2 and S3 showed representative colonies transduced by AAV vectors. Other cells which did not get transduced by AAV vectors can be visualized by phase-contrast images and live fluorescence images for Hoechst-33342 dye.

**Figure 3:**
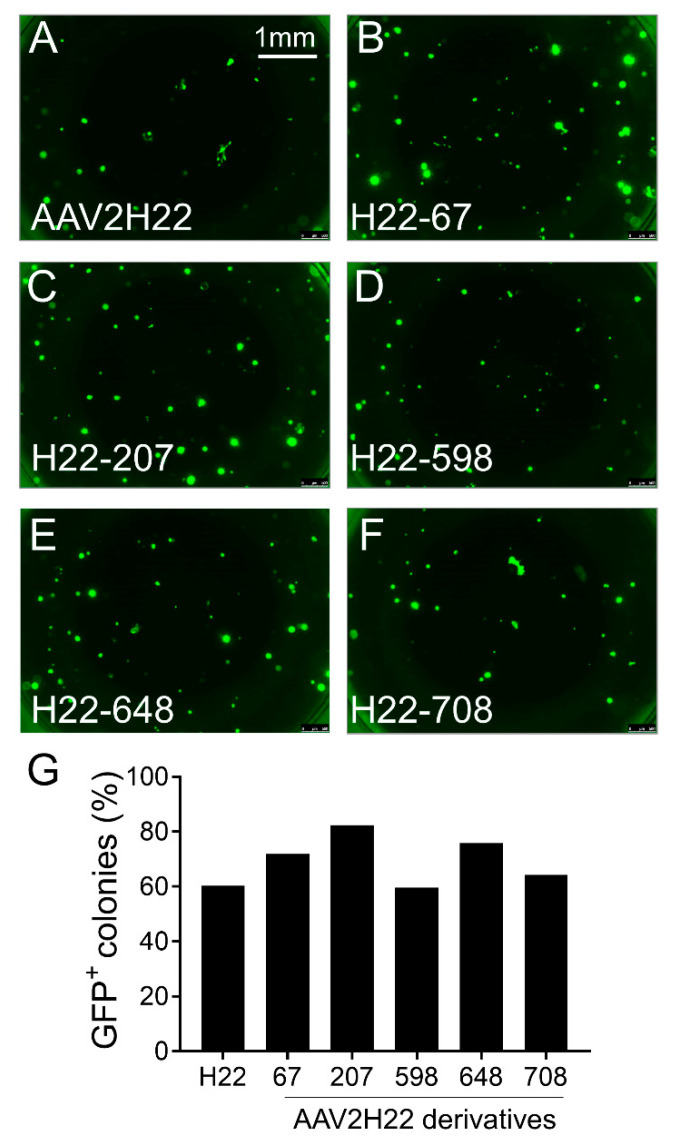
Screening AAV2H22 and derivatives for tropism for ITG α6β4^+^ pig progenitor cells. Freshly isolated ITG α6β4^+^ pig progenitor cells were transduced with the following viral vectors encoding eGFP: AAV2H22 (**A**), AAV2H22 derivatives viral vectors such as AAV2H22-E67A (**B**), AAV2H22-S207G (**C**), AAV2H22-Q598L (**D**), AAV2H22-I648V (**E**), AAV2H22-V708I (**F**). GFP^+^ cluster were quantified after progenitor cells were cultured in the Matrigel for 2 weeks. The percentage of GFP^+^ colonies among the entire progenitor cells for each viral vector were quantified (**G**). All the cells came from non-CF pigs. Scale bars in all images = 1 mm.

**Figure 4:**
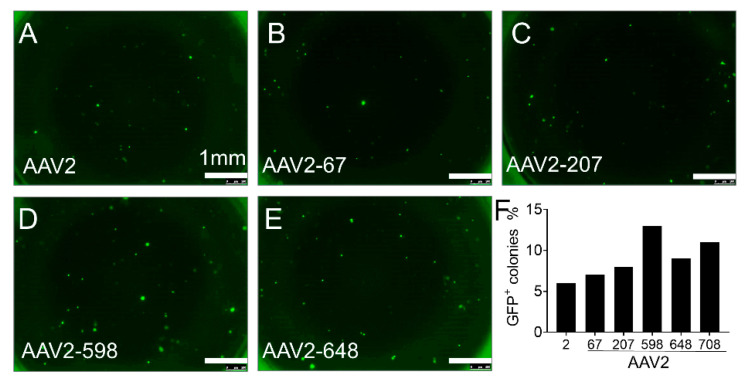
Screening AAV2 and derivatives for tropism for α6β4^+^ pig progenitor cells. Freshly isolated ITG α6β4+ pig progenitor cells were transduced with the following viral vectors encoding eGFP: AAV2 (**A**), AAV2 derivatives viral vectors including AAV2-E67A (**B**), AAV2-S207G (**C**), AAV2-Q598L (**D**), AAV2-I648V (**E**). GFP^+^ clusters were quantified after progenitor cells were cultured in the Matrigel for 2 weeks. Quantification of the percentage of GFP^+^ colonies among the entire progenitor cells for each viral vector shows maximum transduction efficiency was less than 15% (**F**). All the cells came from non-CF pigs. Scale bars in all images = 1 mm.

**Figure 5:**
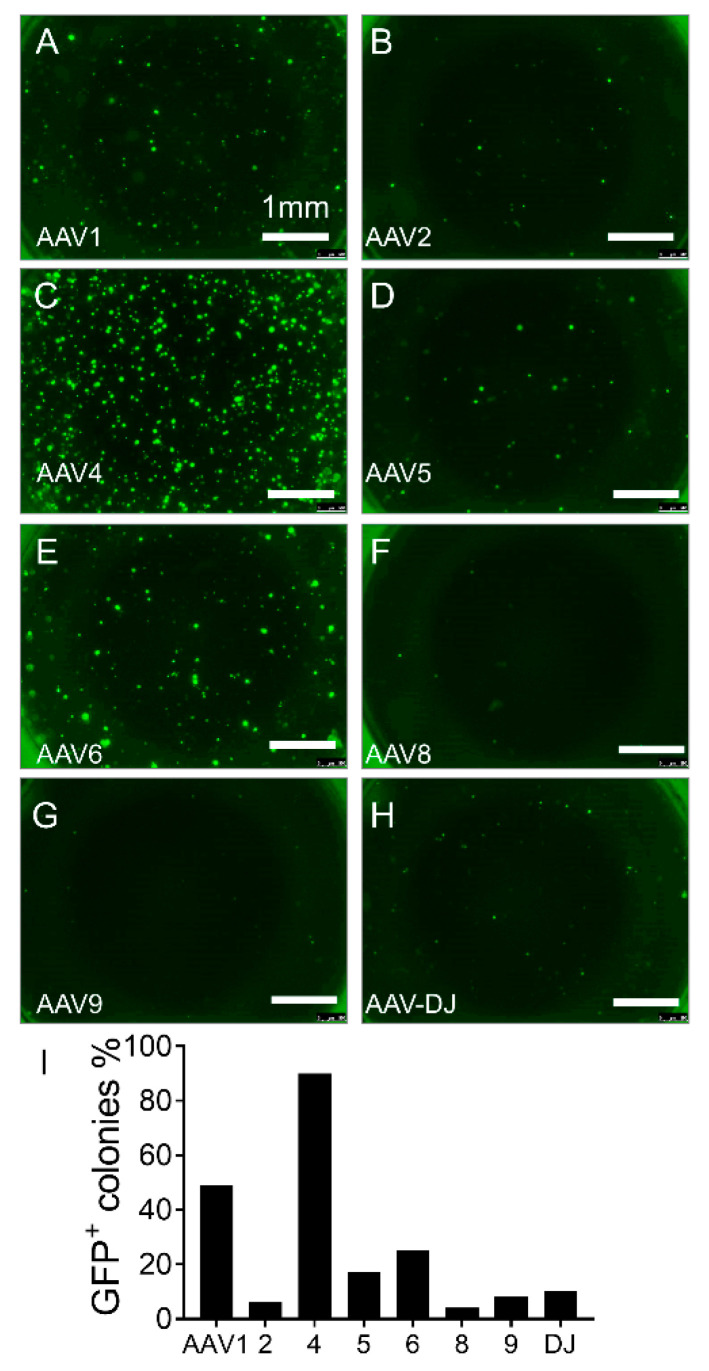
Screening AAV natural serotypes for tropism for α6β4^+^ pig progenitor cells and AAV4 was far superior to all other serotypes. Freshly isolated ITG α6β4+ pig progenitor cells were transduced with the following viral vectors encoding eGFP: AAV1 (**A**), AAV2 (**B**), AAV4 (**C**), AAV5 (**D**), AAV6 (**E**), AAV8 (**F**), AAV9 (**G**), AAV-DJ (**H**). GFP^+^ clusters were quantified after progenitor cells were cultured in the Matrigel for 2 weeks. Quantification of the percentage of GFP^+^ colonies among the entire progenitor cells for each viral vector shows that AAVV4 has the highest transduction efficiency (**I**). All the cells came from non-CF pigs. Scale bars in all images = 1 mm.

We found that AAV2H22 and its derivatives were superior to AAV2 and its derivative with higher transduction efficiency as measured by the percentage of GFP positive DLEPs (Figure 3 and Figure 4). Finally, we screened natural serotypes AAV1, 2, 4, 5, 6, 8, 9, as well as AAV-DJ. Unexpectedly, we found AAV4 to have the highest transduction efficiency on DLEPs of all serotypes, including AAV2 and AAVH22 (Figure 5). We repeated the screening of all AAV vectors, and the results were presented in Figure S4. AAV4 had highest transduction efficiency among all the screened vectors.

### 3.3. AAV4 Has Higher Tropism for Small Airway Epithelia Than Large Airway Epithelia in Pigs

**Figure 6:**
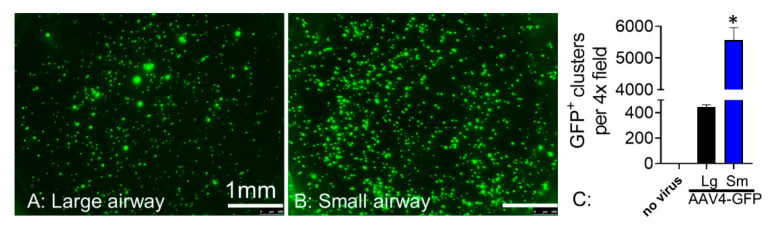
AAV4 transduce both large and small airway epithelial cells but with much higher tropism for small airways. AAV4 encoding eGFP transduces both large (**A**) and small (**B**) pig airway epithelial cells. Scale bars = 500µm. (**C**) Quantification of AAV4-GFP transduced GFP^+^ clusters demonstrate much higher expression in epithelial cells from pig small airways compared with large airways. *N* = 5, * *p* < 0.05 compared to large airways cells. All the cells came from non-CF pigs. Scale bars in all images = 1 mm.

We next expanded studies to assess if AAV4 has tropism for other regions of the pig airways. We transduced primary large and small airway epithelia with AAV4-eGFP and quantified GFP^+^ cells by manual counting two weeks later. AAV4 transduced both large and small airway epithelia (Figure 6). However, the relative transduction efficiency of AAV4 was about 10-fold higher for small airways compared with large airways, as measured by GFP^+^ cells (Figure 6).

### 3.4. Surface Carbohydrate Groups Are Differentially Expressed on Small Airway and Large Airway Epithelia in Pigs

**Figure 7:**
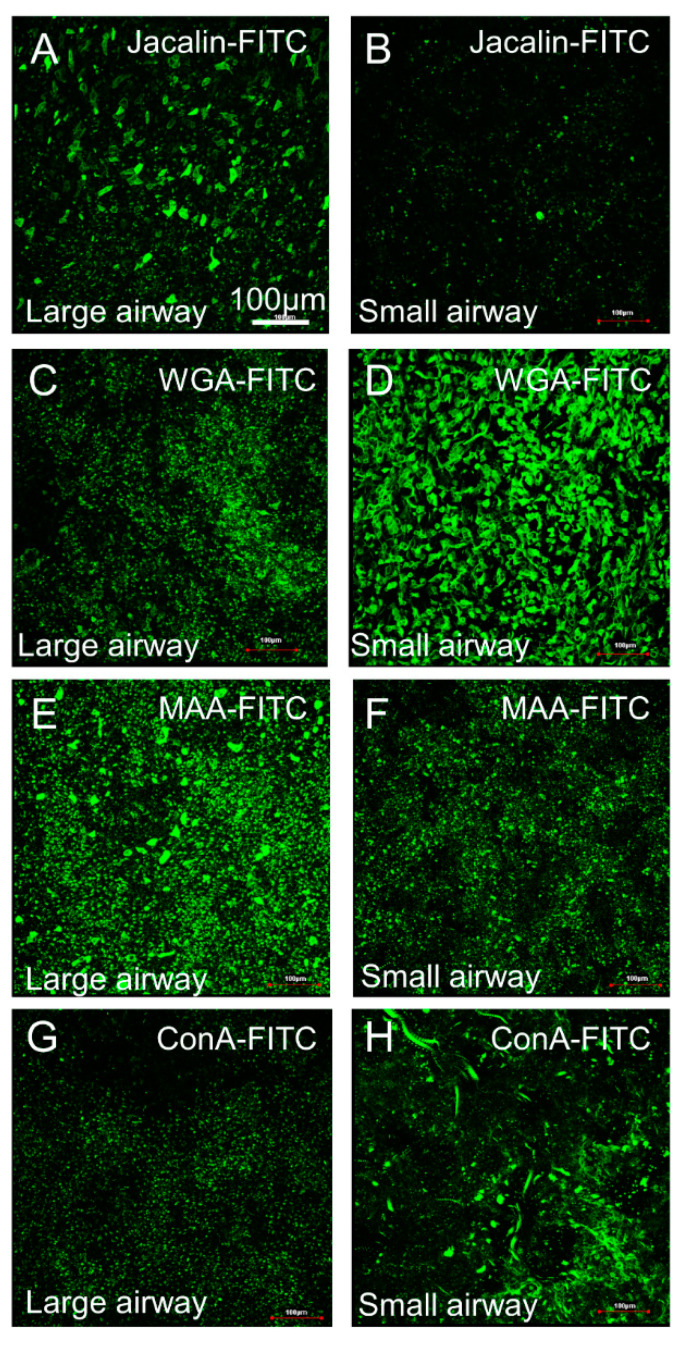
Lectin binding profiles assays in large and small porcine airway epithelium. There is much higher expression of O-linked carbohydrates and O-glycoproteins demonstrated by fluorescence assay for jacalin (**A**,**B**) in large airways compared with small airways. In contrast, there is much higher expression of N-acetyl-D-glucosamine demonstrated by wheat germ agglutinin assay (WGA, (**C**,**D**)) in small airways compared to large airways. In addition, there is more 2,3-linked sialic acid demonstrated by MAA (**E**,**F**) fluorescence assay in large airways compared to small airways. Moreover, there is more mannose demonstrated by ConA (**G**,**H**) fluorescence assay in small airways compared to large airways. All the cells came from non-CF pigs.

In order to investigate the molecular mechanism underlying the higher tropism of AAV4 for small airways compared to large airways, we performed lectin profile staining to detect the expression of specific groups of carbohydrates. O-linked α2,3 sialic acid is found on epithelial surface mucins, has been shown to bind AAV4, and suggested to prevent AAV4 apical entry into human airway cells [28]. Compared with large airways, porcine small airways express much less O-linked carbohydrates/O-glycoproteins and 2,3-linked sialic acid as demonstrated by fluorescence staining with jacalin and MMA, respectively (Figure 7). However, we found much higher expression levels of α-mannose and N-acetyl-D-glucosamine sialic acid in small airways compared to large airways, after staining with ConA wheat germ agglutinin (WGA), respectively (Figure 7). These distinct lectin binding profiles may contribute to the differential transduction efficiencies of AAV4 on small compared with large airways in pigs.

### 3.5. AAV4-CFTR Transduction of CF Pig Small Airway Epithelia Increases CFTR Activity and Expression

**Figure 8:**
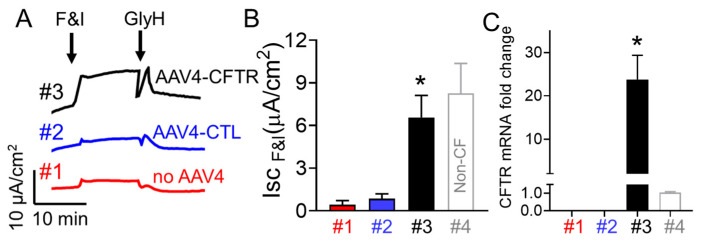
AAV4 encoding CFTR significantly increased cAMP stimulated short circuit current (Isc) and CFTR gene expression in CF pig small airway epithelia. (**A**) Sample Ussing chamber traces from small airway epithelial cells from CF pigs in response to indicated chemical treatments. Trace #1: control without AAV4 treatment; #2: CF cells treated with AAV4 encoding GFP; #3: CF cells treated with AAV4 encoding CFTR. (**B**) Summary of cAMP stimulated Isc. *N* = 3, * *p* < 0.05 compared to #1 and #2. #4, non-CF cells. (**C**) AAV4 encoding CFTR increased CFTR expression in CF cells. *N* = 3. * *p* < 0.05 compared to #1 and #2. #4, non-CF cells.

AAV packaging limitations prohibit cloning the full length CFTR cDNA and key regulatory domains into a recombinant AAV. Hence, a shortened AAV expression cassette for CFTR gene transfer to airway epithelia was developed which contains an optimized truncated CMV promoter and polyA sequence along with a CFTR cDNA lacking residues 708–759 of the regulatory domain (CFTRΔR) [37]. This CFTR variant shows normal biosynthesis, apical membrane targeting, and Cl^−^ channel activity [37]. We have generated an AAV4 vector encoding pig CFTR with R domain deletion controlled by the partial CMV promoter (AAV4-CMV > pCFTR). We transduced cultured CF small airway epithelia with AAV4-CMV > pCFTR or AAV4-CMV-eGFP to determine if AAV4 can be used as a delivery vector for CFTR. Then, two weeks after viral transduction, AAV4 encoding CFTR significantly increased cAMP stimulated short-circuit current (Isc) and CFTR gene expression in CF pig airway epithelia in vitro (Figure 8), demonstrating that AAV4 can be used as a delivery vector for CFTR for CF gene therapy.

### 3.6. In Vivo Airway Administered AAV4 Targets the Distal Small Airways in Pigs 

**Figure 9:**
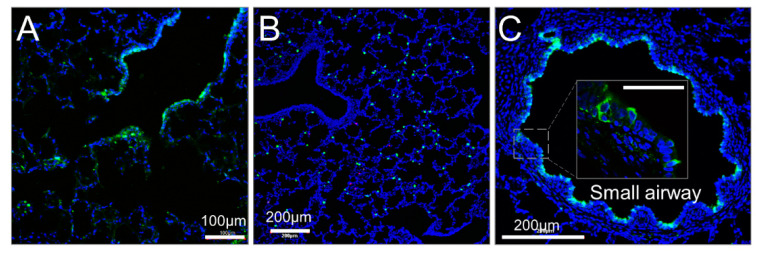
AAV4-mediated transgene expression in pig distal lung. AAV4-CMV > eGFP was administered via bronchoscope to pig distal lung. Immunofluorescent imaging of tissue confirmed expression of GFP^+^ cells in bronchioalveolar junction (**A**), alveolar region (**B**), and small airways (**C**) in vivo. All the images came from non-CF pigs. *N* = 3.

Delivering transgenes in vivo has been a central challenge to gene therapy for CF lung disease. Pig lungs are anatomically and structurally similar to human lungs, and pigs are an ideal preclinical and translational model for CFTR gene therapy [15]. To explore the feasibility of viral delivery to the small airway in vivo, we monitored the distribution of CT imaging contrast agent instilled via bronchoscope to the left lung. Analysis of images from different view demonstrated that the contrast agent reached to the distal left lung, with broad distribution (Figure S5). We assessed the efficiency of cell targeting by AAV4 encoding eGFP following in vivo viral delivery to farm pigs. AAV4-CMV > eGFP was administered by airway instillation to both the trachea and distal lung. Then, two weeks later, lung tissue was fixed for immunofluorescent imaging. GFP transferred from AAV4 was detected in cells harvested from the small airways and the bronchoalveolar junction area, but rarely in the large airways (Figure 9). These results suggest that AAV4 has much higher tropism for small airways than for large airways. The ability of AAV4 to deliver target genes to small airways which play an important role in early CF pathogenesis urges future preclinical studies in CF pigs in vivo, and potential translational studies in humans.

## 4. Discussion

Our analysis of AAV natural serotypes using cultured primary pig airway epithelia showed that AAV4 has high tropism for pig airway epithelia, with higher transduction efficiency in small airways compared to large airways. The higher efficiency in small airways is likely due to decreased mucin expression there, and because the different sialic acids (such as α-mannose and *N*-acetyl-d-glucosamine sialic acids) glycosylated mucins does not inhibit AAV4 transduction there [6,40,41]. Previous studies also reported that the linkage and distribution of sialic acid are vastly different in pig large and small airways [42].

Moreover, AAV4 is part of an evolutionary AAV lineage that does not require AAV receptor [43], and we have shown that vectors capable of AAV receptor-independent infection more efficiently transduce airway epithelia from apical side [44]. In addition, AAV4-mediated delivery of CFTR increased Cl^−^ transport in CF pig small airway epithelium in vitro. Hence AAV4, with much higher tropism for small airway epithelia compared with large airways, will enable us to mechanistically investigate what has been suggested by clinical findings, namely that small airways play an important role in CF pathogenesis [7].

Our study focuses on small airways, which are likely the key sites of CFTR expression due to distinct features including increased CFTR activity and higher pH of air surface liquid compared with large airways [6]. Clinical studies also suggest small airways are a key region involved in the early manifestations of CF lung disease. For example, in CF children the FEF 25–75 marker of small airway obstruction usually declines before large airway FEV1 indications. Yet, due to the inaccessibility of small airways in CF patients, there are currently few published studies investigating how CFTR affects small airways. Moreover, an CF animal model recapitulating human anatomy and physiology beginning at birth has only recently become available, with the development of the CF pig model by our group.

Progress has been made in developing CFTR correctors and potentiators to treat many people with CFTR mutations, but there has been no therapy to restore CFTR function irrespective of the mutations [3,45,46,47]. Efficient targeted delivery of CFTR cDNA to CF lungs might correct the CFTR defect, restore host defense mechanisms, and prevent lung infections [15,48]. The CF pig model provides a new, and uniquely valuable opportunity to investigate novel viral-based gene therapy approaches to prevent CF lung disease.

CF pigs develop the characteristic manifestations of human CF, including abnormalities of the pancreas, lung, intestine, liver, and other organs. Moreover, spontaneous bacterial airway infections emerge in CF pig lungs [2]. We have detected host defense defects in both large and small airways in CF pigs, at least partially due to ASL pH dysregulation. Compared to non-CF animals, the large airways of newborn CF pigs have a reduced airway surface liquid (ASL) pH in vivo, ex vivo, and in differentiated primary cultures of airway epithelia. These findings prompted us to investigate the nasal pH in newborn babies with CF, where we found nasal ASL was also more acidic than in non-CF neonates. Moreover, the ATP12A proton pump acidifies the large airways, and lack of ATP12A expression in CF mice may explain, at least in part, the lack of a lung phenotype [49]. In our recent studies, the ASL from CF distal small airways were nevertheless more acidic compared to non-CF although porcine small airway tissue does not express ATP12A (like mice). We further found that small airways express ATP6V0D2, an isoform of the V0d subunit of the H^+^-translocating plasma membrane V-type ATPase to the apical surface only when the pH is alkaline [50]. Similar to the large airways, CF small airways exhibit a defect in mucociliary clearance only detectable when stimulated to secret mucus. Future work using AAV4 to restore CFTR function or knockdown ATP6V0D2 function in CF small airways will facilitate mechanistic studies of small airway biology ex vivo or in vivo, and open up novel avenues for CF treatments.

Additionally, the knowledge generated by this study will allow AAV4 to be used as a research tool in pigs to develop models for lung diseases apart from CF. The advantages of using pigs as preclinical models include: (1) The pig lung epithelial ion composition is more similar to those found in human lungs than are rodent compositions; (2) Pig organs share many anatomical, histological, physiological, and biochemical responses with human organs and have been used in biomedical research for multiple human diseases [51]; (3) The body size of a pig provides the opportunity to collect enough samples, as well as obtain adequate lung computed tomography (CT) imaging resolution. Hogg et al. [52,53] have shown that the destruction of small airways precedes the development of emphysema, AAV4-mediated suicide gene delivery to small airways in pigs could determine whether induced-small airway injury is causative for COPD-like lung diseases. Moreover, AAV4 was superior to all other AAV natural serotypes in transducing ITGα6β4^+^ pig distal lung progenitor cells and will be a useful tool to study pathogenesis of common pig respiratory diseases, such as swine influenza.

## Data Availability

The data presented in this study are available on request from the corresponding authors.

## Supplementary Materials

The following are available online at https://www.mdpi.com/article/10.3390/cells10051014/s1, Figure S1: Schematic workflow for isolation of large, small airway epithelial cells and distal lung progenitor cells from pig lungs. Figure S2: Transduction of distal lung progenitor cells by AAV vectors. Figure S3: Transduction of distal lung progenitor cells by AAV vectors. Figure S4: The second run of screening of AAV vectors for tropism for ITG α6β4+ pig progenitor cells. Figure S5: The feasibility of delivery of AAV vectors to small airways in vivo.

## Abbreviations

The following abbreviations are used in this manuscript:

AAV4	Adeno-associated virus 4
CF	Cystic fibrosis
CFTR	CF transmembrane conductance regulator
FEV1	Forced Expiratory Volume in 1 s
COPD	Chronic Obstructive Pulmonary Disease
DLEPs	Distal lung epithelial progenitors
ITG	Integrin
FACS	Fluorescence-activated cell sorting
ConA	Concanavalin A
MMA	Maakia amurensis lectin
