# Supplementary material for: Transduction of Pig Small Airway Epithelial Cells and Distal Lung Progenitor Cells by AAV4
Source: Cells. 2021 Apr 25;10(5):1014. doi: 10.3390/cells10051014 (PMC8145967; doi:10.3390/cells10051014)
Supplement: Supplementary file 1 [file cells-10-01014-s001.zip › cells-1182847-supplementary.pdf]

1. Supplementary Materials

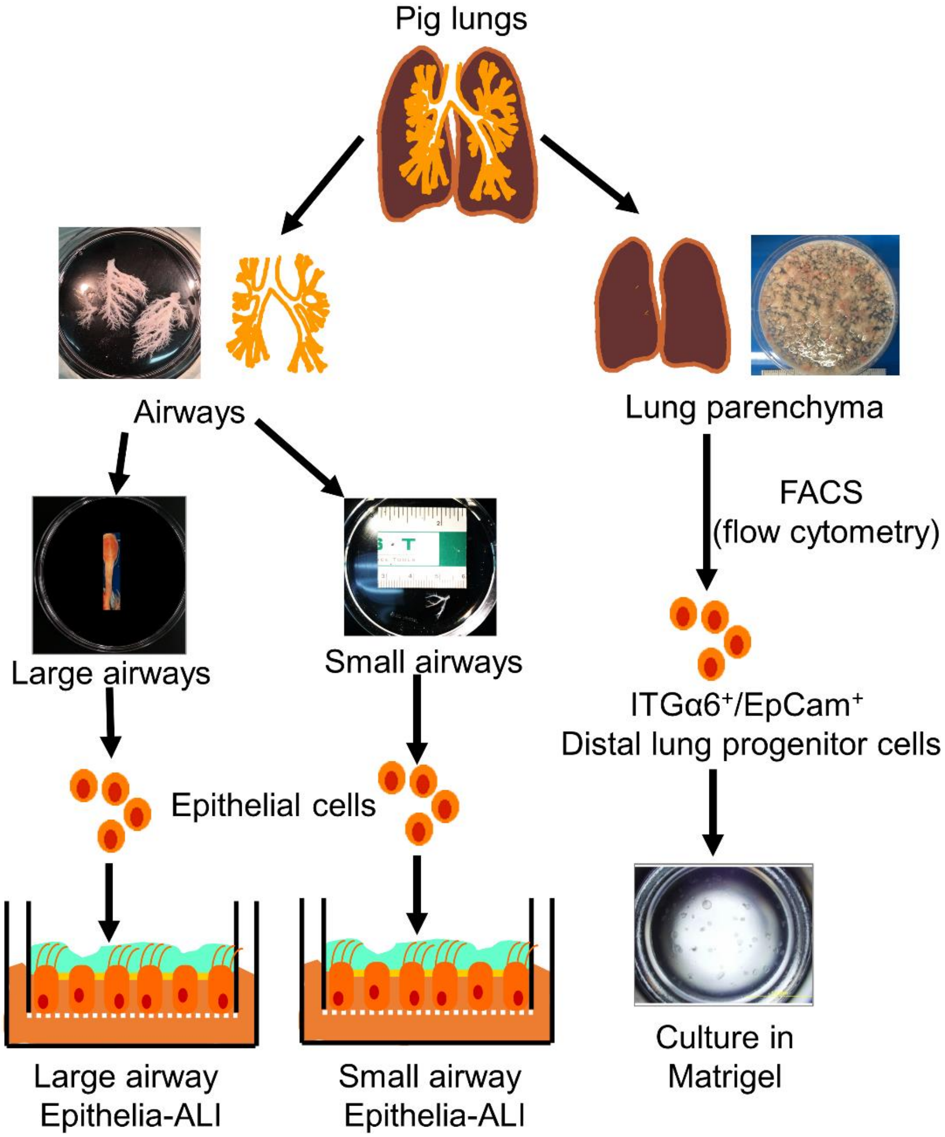

Figure S1. Schematic workflow for isolation of large, small airway epithelial cells and distal lung progenitor cells from pig lungs. First, airways were detached from lung parenchyma by blunt dissection. Then small airways were further separated from large airways by microdissection. Large, small airways cells and distal lung progenitor cells were isolated from different regions of the pig lungs. Large and small airways cells were cultured in transwells at the air-liquid interface (ALI). DLEPs were cultured in Matrigel for 2 weeks.

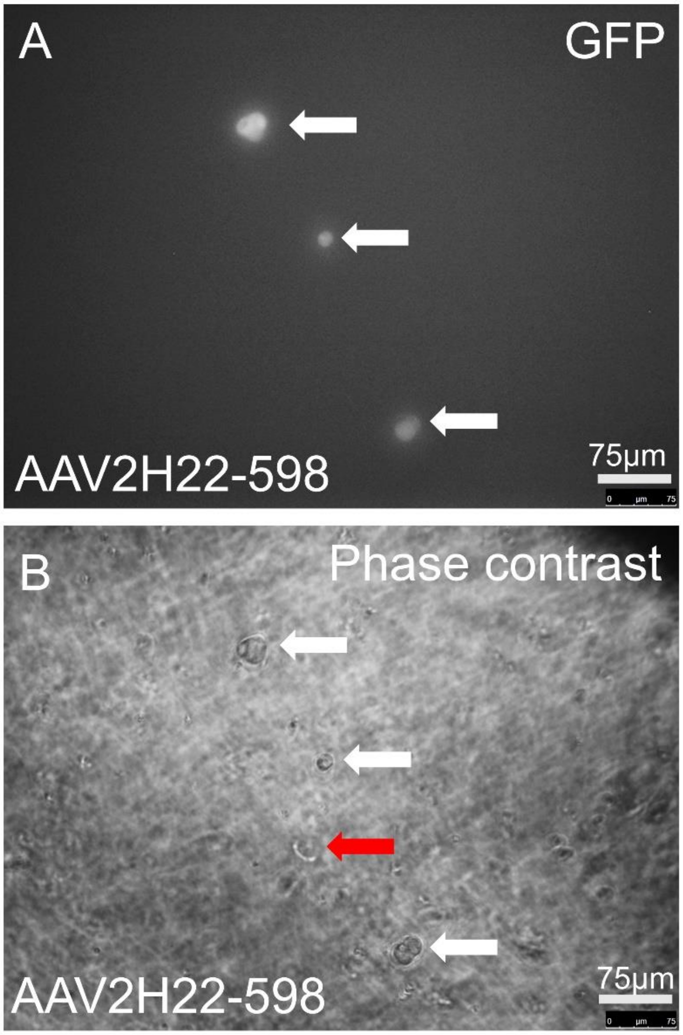

Figure S2. Transduction of distal lung progenitor cells by AAV vectors. Freshly isolated ITGα6β4+ pig progenitor cells were transduced with the AAV2H22-Q598L viral vector encoding eGFP. The DLPEs formed colonies in the Matrigel 2 weeks after seeding. (**A**) GFP$^+$ colonies were transduced by viral vector (white arrows). (**B**) Phase contrast images showed that all colonies and other cells did not form colonies. Note the colony pointed by red arrow did not get transduced by AAV vector. Scale bars in all images = 75 µm.

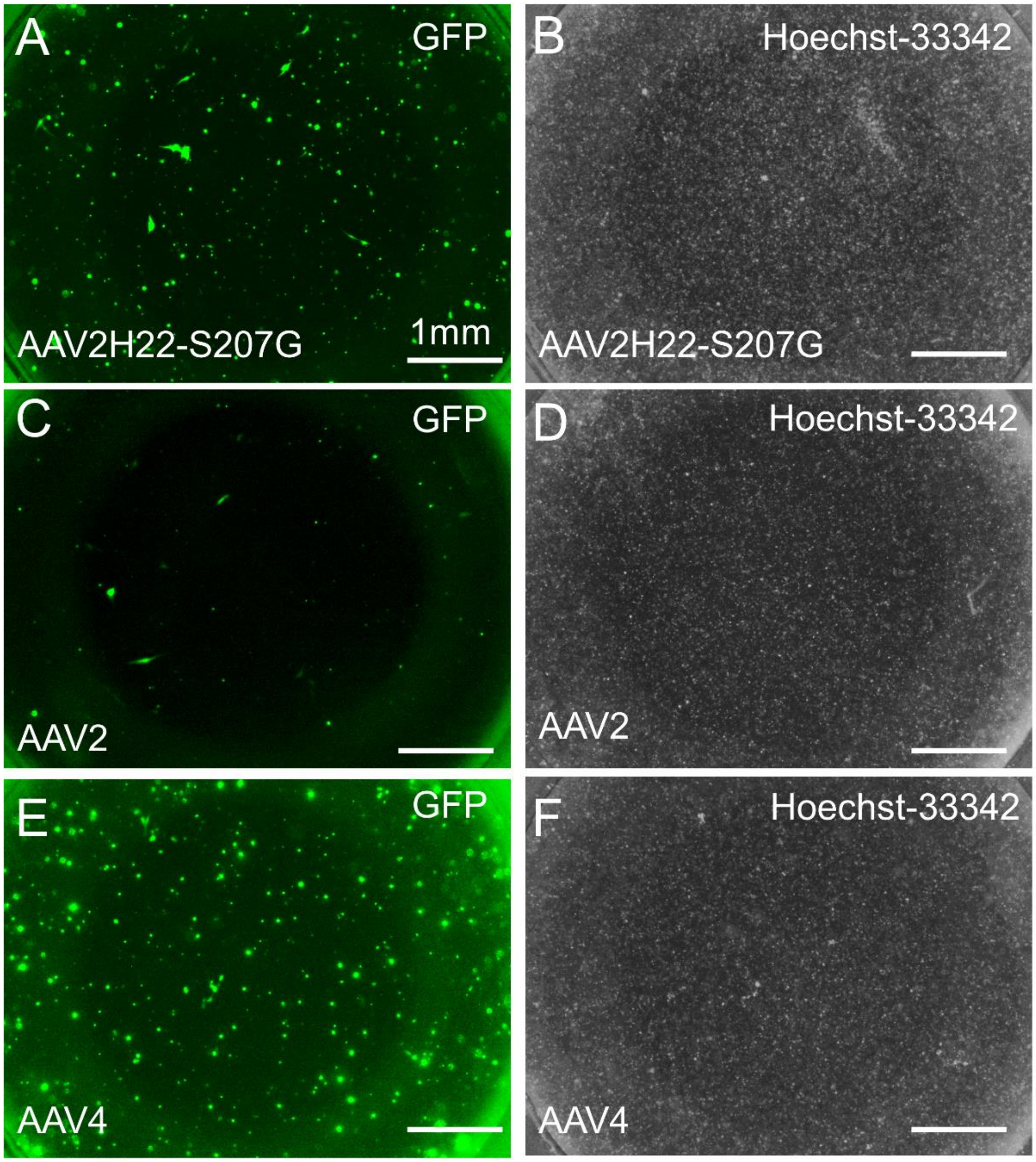

Figure S3. Transduction of distal lung progenitor cells by AAV vectors. Freshly isolated ITGα6β4+ pig progenitor cells were transduced with the AAV2H22-S598G, AAV2 and AAV4 viral vectors encoding eGFP. The DLPEs formed colonies in the Matrigel 2 weeks after seeding. (**A**) GFP+ colonies were transduced by viral vector (**A**, **C** and **E**). Live fluorescence images (**B**, **D** and **F**) to show all the cells stained by Hoechst-33342 dye in the corresponding transwells. Scale bars in all images = 1 mm.

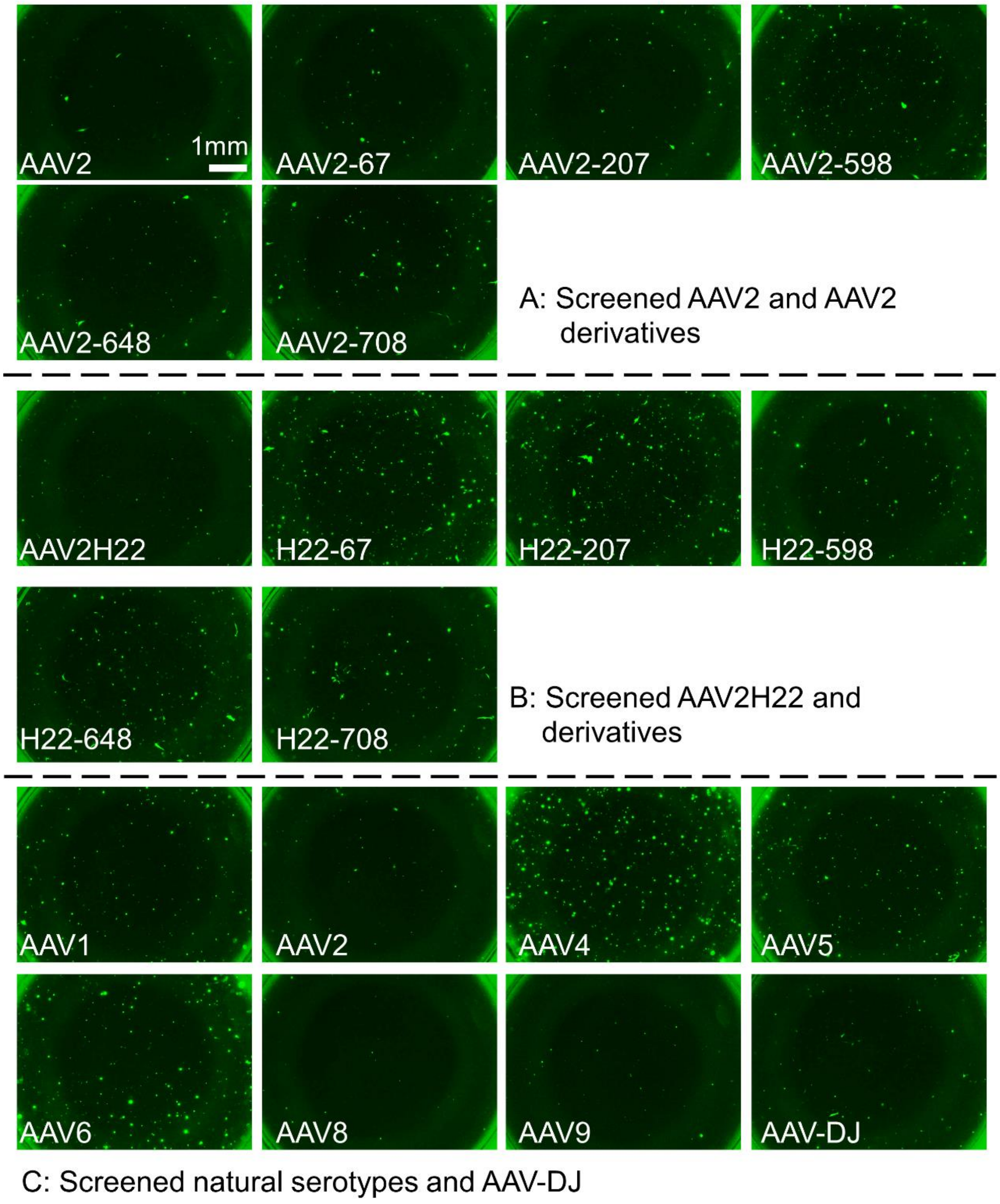

Figure S4. The second run of screening of AAV vectors for tropism for ITG $\alpha6\beta4^{+}$ pig progenitor cells. (**A**) Screened AAV2 and derivatives. (**B**) Screened AAV2H22 and derivatives. 3 Screened natural serotypes and AAV-DJ. AAV4 had highest transduction efficiency among all the screened vectors. Scale bar in all images = 1 mm.

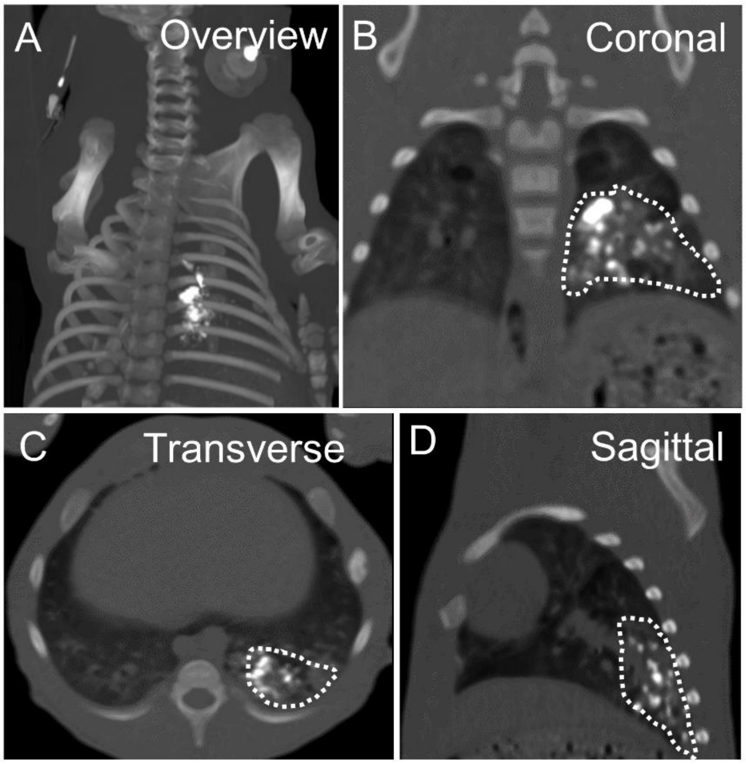

Figure S5. The feasibility of delivery of AAV vectors to small airways *in vivo*. CT imaging contrast (0.5 mL) was instilled via bronchoscope to the left lung of a 1-week old farm pig. CT images from different views (**A**–**D**) demonstrated that it reached to the distal left lung with broad distribution (dotted lines).

2. CT imaging

To test the feasibility of AAV4 delivery to the distal lung, we used a previously described X-ray computed tomographic (CT) based assay [1]. Animals were anesthetized, intubated, and CT imaging contrast (0.5 mL) was instilled via bronchoscope to the left distal lung. Immediately after delivery, the catheter was removed. CT scans were acquired with a high-resolution multi-row detector computerized tomography scanner (Siemens Somatom, Definition Flash Dual Source 128-slice computed tomography scanner) as previously described [1]. A series of CT scans were obtained. Distribution of the imaging contrast for different positions was determined.

Reference

1. Hoegger, M.J.; Fischer, A.J.; McMenimen, J.D.; Ostedgaard, L.S.; Tucker, A.J.; Awadalla, M.A.; Moninger, T.O.; Michalski, A.S.; Hoffman, E.A.; Zabner, J. et al. Impaired mucus detachment disrupts mucociliary transport in a piglet model of cystic fibrosis. *Science* **2014**, *345*, 818–822.
